# Changes in the sociodemographic composition of the lowest socioeconomic group over time, 1987–2001

**DOI:** 10.1186/1471-2458-7-305

**Published:** 2007-10-25

**Authors:** Frans JM Bongers, Linelle EN Deunk, Francois G Schellevis, Henk JM van den Hoogen, Jouke van der Zee, Wil JHM van den Bosch

**Affiliations:** 1 Netherlands Institute for Health Services Research NIVEL, Utrecht, The Netherlands; 2 University Medical Centre st Radboud Nijmegen, Department of General Practice 229 HSV, Postbus 9101, 6500 HB Nijmegen, The Netherlands

## Abstract

**Background:**

When comparing health differences of groups with equal socioeconomic status (SES) over time, the sociodemographic composition of such a SES group is considered to be constant. However, when the periods are sufficiently spaced in time, sociodemographic changes may have occurred. The aim of this study is to examine in which respects the sociodemographic composition of lowest SES group changed between 1987 and 2001.

**Methods:**

Our data were derived from the first and second Dutch National Survey of General Practice conducted in 1987 and 2001. In 1987 sociodemographic data from all listed patients (N = 334,007) were obtained by filling out a registration form at the practice (response 78.3%, 261,691 persons), in 2001 these data from all listed patients (385,461) were obtained by postal survey (response 76.9%, 296,243 persons). Participants were primarily classified according to their occupation into three SES groups: lowest, middle and highest.

**Results:**

In comparison with 1987, the lowest SES group decreased in relative size from 34.9% to 29.5%. Within this smaller SES group, the relative contribution of persons with a higher education more than doubled for females and doubled for males. This indicates that the relation between educational level and occupation was less firmly anchored in 2001 than in 1987.

The relative proportion of some disadvantaged groups (divorced, unemployed) increased in the lowest SES group, but the size of this effect was smaller than the increase from higher education. Young people (0–24 years) were proportionally less often represented in the lowest SES group.

Non-Western immigrants contributed in 2001 proportionally less to the lowest SES group than in 1987, because of an intergenerational upward mobility of the second generation.

**Conclusion:**

On balance, the changes in the composition did not result in an accumulation of disadvantaged groups in the lowest SES group. On the contrary, the influx of people with higher educational qualifications between 1987 and 2001 could result in better health outcomes and health perspectives of the lowest SES group

## Background

Before 1980 not much attention was paid to socioeconomic inequities in health. Most believed that the health differences related to socioeconomic differences would decrease by spreading of welfare and by achieving equal accessibility to the health care system for all. This changed in the early 1980's because of the publication of the Black Report in England [1]. Socioeconomic health inequities became a major political and public concern also in the Netherlands with a focus on health education and health care provision in disadvantaged subgroups [2].

When comparing health differences of groups with equal socioeconomic status (SES) over two time periods, the sociodemographic composition of such a SES group is considered to be constant. However, when the two periods are sufficiently spaced in time, sociodemographic changes may have occurred.

The aim of this study is to examine in which respects the sociodemographic composition of lowest SES group changed between 1987 and 2001. This is an important issue because changes in composition may affect health outcomes and health perspectives of the lowest SES group. For the purpose of this study we divided the socioeconomic spectrum in three groups: the lowest, the middle and the highest SES group.

The most utilized socioeconomic indicators are level of education, occupation and income. These three dimensions of SES are strongly related and complementary, but not interchangeable [3]. Each indicator is likely to reflect both common impacts of a general hierarchical ranking in society as well as particular impacts specific to the indicator [4].

A lower socioeconomic status influences health in an unfavourable way through the presence of unhealthy lifestyle factors, unequal access to – and quality of – health care, more material deprivation and a stressful psychosocial environment [5].

Volkers et al. found that a low occupational position was consistently associated with poor health and physician-diagnosed morbidity, which could not be explained by a low educational level [6].

Theoretically a combination of measures for deriving socioeconomic status would be preferable [4], but on practical grounds most often a single item is used for measuring socioeconomic status.

There is no single best indicator of SES suitable for all study aims and applicable at all time points in all settings. The choice of socioeconomic indicator often reflects which data are available rather than any explicit theorisation of the possible effects of different dimensions of socioeconomic disadvantage [7].

In the US education has been widely used as SES indicator, because educational data are most readily available [8], whereas in Britain occupation social class is the more usual measure [7].

In this paper we chose occupation as SES indicator.

We know that between 1987 and 2001 the following changes in sociodemographic composition took place:

- the general educational level in the population increased between 1987 and 2001

- The proportion of non-western immigrants rose between 1987 and 2001 and within the group of non-western immigrants the proportion of persons from the second generation (persons born in the Netherlands but with a at least one parent born outside the Netherlands) grew [9].

Considering these changes, we hypothesise that a higher educational level in the population will lead to better job perspectives and will express itself in a smaller lowest SES group. On the other hand, we expect that the competition on the labour market for higher job categories will be higher, because more people with higher education are available; because of this phenomenon more persons with a higher educational attainment might reside in the lowest SES group. We expect in particular that women are at risk for staying in an occupation-based low SES-group because their job is more likely to be incongruent to their educational level, due to the balancing between family and work.

Younger people might be classified less frequently in the lowest socioeconomic group, because of an increasing educational level.

As far as non-Western immigrants are concerned, we suppose less people will have a low SES, due to an intergenerational upward mobility of the second generation.

We formulated the following research questions.

1) What is the size and direction of changes of the different SES-groups between 1987 and 2001?

2) What is the difference in educational attainment in males and females of the lowest SES group when comparing 1987 with 2001?

3) What is the difference in the composition of the lowest SES-group of other sociodemographic determinants when comparing 1987 with 2001?

## Methods

### Study population

In the Netherlands, the entire non-institutionalised population is registered at a general practice. Patients enlisted in the practices participating in the first and second Dutch National Survey of General Practice (DNSGP-1 and DSNGP-2) were used as our study populations. Data collection for these studies took place in 1987 and 2001; in 103 (161 general practitioners) and 104 (195 general practitioners) practices respectively. In both surveys a representative sample of practices and the Dutch population was used. All patients listed in the participating practices were included creating study populations of 334,007 (DNSGP-1) and 385,461 (DNSGP-2) persons.

### Data collection

Data required for this study were obtained from patient registration forms [10,11]. Sociodemographic data from all listed patients (Table 1) were collected by filling out a registration form via the practice (1987) or at home via the postal survey (2001); ìn 1987 78.3% of these patients responded in a sociodemographic census, in 2001 76.9% of the patients responded (Table 1). Age and gender did not differ between responders and not-responders.

**Table 1:** Characteristics of total population compared with respondents in 1987 and 2001

	1987		2001	
	All (N = 334,007)	Respondents N = 261,691	All (N = 385,461)	Respondents (N = 296,243)
	%	%	%	%
**SEX**				
male	49.5	49.0	48.2	48.8
female	50.5	51.0	51.8	51.2
**Age (yrs)**				
0–24 y	36.5	33.0	29.7	29.4
25–64	51.5	55.0	57.1	56.1
65 y a.o	12.1	12.0	13.2	14.4
**Marital status**				
unmarried	44.9	41.0	41.3	41
married	47.2	50.8	49.9	50.3
divorced	2.6	2.8	3.3	3.2
widowhood	5.2	5.4	5.5	5.5
**Household composition**				
single household	10.3	9.7	12.7	12.5
couple	21.1	21.4	29.3	29.4
single parent family	4.9	4.6	5.9	5.8
couple with children	63.8	64.3	52.0	52.3
**Employment status**				
pupil/student	31.7	28.7	22.8	22.4
paid job	34.4	36.9	44.0	44.3
unemployed	2.4	2.4	1.6	1.6
disabled	13.3	14.4	15.2	15.2
housewife/man	2.9	3.1	4.2	4.2
retired	15.4	14.4	12.2	12.3
**Ethnic background**				
native	91.9	92.0	87.7	87.8
western immigrant	5.2	5.3	6.2	6.2
non-western immigrant	2.9	2.7	6.1	6.0
**SES groups **(%)				
Lowest		34.9		29.5
Middle		48.6		42.4
Highest		16.5		28.8

Representativeness was kept in both studies for sex, age, and type of health insurance. Data collection procedures and instruments towards socio-demographic data were identical to ensure good comparability.

### Socioeconomic status

Occupation was used as the socioeconomic indicator. Participants were asked to fill in their last occupation instead of their current occupation. This has the advantage that also persons were included, who were unemployed, retired or disabled.

The registered occupation was coded according to the Standard Classification of Occupations (SBC92 of Statistics Netherlands [12]), which is strongly related to the International Standard Classification of Occupation (ISCO88) [13]. Participants were classified according to their occupation into three SES groups: lowest, middle and highest.

In the highest SES group the managerial en professional occupations were placed; in the middle SES group the small employers, own account workers and intermediate occupations (clerical, administrative, sales workers with no involvement in general planning or supervision); and in the lowest SES group people in lower supervisory and technical occupations, and (semi) routine occupations.

In 1987 68.9% of the respondents were classified in one of the three SES groups according to their occupation, 21.4% according the highest occupational level of the household and 9.7% according to the highest educational level; in 2001 57.5% was classified according to occupation, 27.3 according to the highest occupational level of the household and 15.2% according to the highest educational level.

Because the educational level was used for assigning the socioeconomic status in a part of the respondents, we excluded in the analysis of the relation between socioeconomic status and educational attainment those persons, whose classification in the lowest SES group was based on educational level. In this way 14,445 respondents were left out in 1987 and 19638 respondents in 2001.

### Sociodemographic variables

The following sociodemographic variables were included with between brackets the subdivision in separate socio-demographic subgroups: sex (male-female), age (age groups), marital status (married, unmarried, divorced, widowhood), household composition (couple with children, couple, single household, single parent family), employment status (student, paid job, unemployed, housewife/man, disabled for work, retired), ethnic background (based on the country of birth of the respondent and his/her parents categorized as native, western immigrant, non-western immigrant) and highest completed educational level (low, middle, high), and All data were extracted from the patient registration forms.

### Data-analysis

To study changes within the lowest SES group, we determined the distribution in 1987 and 2001 for each sociodemographic category separately across the three SES groups and for all respondents in the population.

For example: in 1987, 33.2 percent of all males were in the lowest SES group, 48.5 percent in the middle SES and 18.3 percent in the highest SES group ; in 2001, 26.3 percent of the males belonged to the lowest SES group (Table 2, column 2 and 3). From these figures we computed for *males *the ratio 2001/1987 (26.3/33.2 = 0.79) of the lowest SES group (column 4). In the same way we calculated the ratio 2001/1987 of the lowest SES group for the *whole population *(29.5% in 2001, 34.9% in 1987: ratio 0.85). This overall ratio of 0.85 was used as reference since we were interested whether the relative proportion of specific sociodemographic categories increased or decreased compared with the overall change. By dividing the "unadjusted" ratio of males by the reference ratio (0.79/0.85), we found the relative change of the proportion of males within the lowest SES group (0.94). We call this the adjusted ratio (column 5). A ratio value above 1.0 indicates a relative increase and a value lower than 1.0 a relative decrease of the proportion for that specific socio-demographic subgroup.

**Table 2:** Change in composition of the lowest SES group 1987–2001*:

	2	3	4	5	6	7	8
	Lowest SES **1987** N = 91,302	Lowest SES **2001** N = 87,525	**Unadjusted** 2001/1987	*Adjusted* *2001/1987/REF*	Proportion in lowest SES group **2001** N = 87,525	Proportion in population of respondents **2001** N = 296,243	low SES/all respondents **2001**
	%	%	ratio	*ratio*	%	%	*Ratio*
**All**	34.9	29.5	0.85	*1.00*	100.0	100.0	*1.00*
**SEX**							
male	33.2	26.3	0.79	*0.94*	43.4	48.8	*0.89*
female	36.5	32.6	0.89	*1.06*	56.6	51.2	*1.11*
**Age (yrs)**							
0–24 y	29.8	19.2	0.65	*0.76*	19.2	29.4	*0.65*
25–64	32.1	30.1	0.94	*1.10*	57.2	56.1	*1.02*
65 y a.o	61.8	48.4	0.78	*0.92*	23.7	14.4	*1.64*
**Marital status**							
unmarried	28.8	22.3	0.77	*0.92*	30.7	41.0	*0.75*
married	36.2	32.2	0.89	*1.05*	54.4	50.3	*1.08*
divorced	36.6	38.9	1.06	*1.26*	4.2	3.2	*1.31*
widowhood	66.4	58.1	0.88	*1.04*	10.7	5.5	*1.95*
**Household composition**							
couple with children	30.8	22.5	0.73	*0.86*	40.5	52.3	*0.77*
single household	46.3	41.4	0.89	*1.06*	17.8	12.5	*1.42*
couple	40.3	33.6	0.83	*0.99*	33.9	29.4	*1.15*
single parent family	40.0	38.8	0.97	*1.15*	7.8	5.8	*1.34*
**Employment status**							
pupil/student	25.7	15.6	0.61	*0.72*	11.7	22.4	*0.52*
paid job	27.9	27.7	0.99	*1.17*	41.0	44.3	*0.93*
unemployed	43.8	42.2	0.96	*1.14*	2.2	1.6	*1.38*
disabled	55.8	46.3	0.83	*0.98*	6.5	4.2	*1.55*
housewife/man	39.5	43.1	1.09	*1.29*	21.9	15.2	*1.44*
retired	59.9	40.7	0.68	*0.80*	16.7	12.3	*1.36*
**Ethnic background**							
native	34.0	28.6	0.84	*1.00*	85.2	87.8	*0.97*
western immigrant	31.5	28.0	0.89	*1.05*	5.9	6.2	*0.95*
non-western immigrant	58.2	44.0	0.76	*0.89*	8.9	6.0	*1.48*
**Education males**	N = 34,881	N = 32,241			N = 32,241	N = 126,917	
(not) yet	25.9	15.9	0.61	*0.73*	11.4	18.3	*0.63*
low	67.5	43.3	0.64	*0.76*	26.0	15.3	*1.70*
middle	22.2	30.8	1.39	*1.64*	58.2	48.0	*1.21*
high	3.1	6.1	1.97	*2.33*	4.4	18.5	*0.24*
**Education females**	N = 37,345	N = 31,863			N = 31,863	N = 112,475	
(not) yet	26.3	15.0	0.57	*0.67*	10.0	19.0	*0.53*
low	71.3	45.9	0.64	*0.76*	22.5	13.9	*1.62*
middle	19.3	35.7	1.85	*2.19*	63.0	50.0	*1.26*
high	2.9	7.2	2.50	*2.96*	4.4	17.1	*0.26*

* Column 2 and 3 the percentage of the lowest SES group in relation to all respondents; Column 4 the ratio 2001/1987 of each sociodemographic subgroup, Column 5 the ratio of each sociodemographic subgroup compared with the overall ratio of 0.85; Column 6 and 7 the proportional distribution of sociodemographic subgroups within each variable for the lowest SES group and for all respondents; Column 8 the ratio lowest SES group/all respondents (column 6/7)

The consequences of all these changes on the lowest SES group in 2001 is shown in column 6 to 8 of Table 2; the proportional distribution of sociodemographic subgroups within each variable is given for the lowest SES group and for all respondents of the population. This difference is in column 8 expressed as a ratio (low SES/all respondents).

## Results

### Socioeconomic status

The relative size of the SES groups changed during 1987–2001 as shown inTable 1: the relative size of the lowest SES group decreased from 34.9% to 29.5% of the population; the relative size of the middle SES group declined from 48.6% to 42.4%; the relative size of the highest SES group increased from 16.5 to 28.2%. This shift between the three SES groups is highly statistically significant (p < 0.001).

The 2001–1987 ratio of the lowest SES group was 0.85 (29.5/34.9), the ratio of the middle SES group 0.87 and of the highest SES group 1.72. We elaborate further on the results of the lowest SES group.

### Lowest SES group in 1987 and 2001

For each sociodemographic subgroup, the proportion with a low SES in 1987 and 2001 are shown in Table 2 in column 2 and 3, whereas column 4 and 5 show the ratio's derived from these columns.

The adjusted ratio in column 5 is an indication for the change of each category between 1987 and 2001 compared with the overall change of 0.85 (used as reference).

Compared with the general trend, the relative proportion of people with the following characteristics *increased *most in the lowest SES group:

• the highest and middle educational group in females (2.96 and 2.19)

• the highest and middle educational group in males (2.33 and 1.64)

• housewife/man (1.29)

• divorced (1.26)

On the other hand, a *decrease *in the relative proportion was seen in

• males and females with not yet accomplished education (0.73 and 0.67)

• males and females with low educational level (0.76 and 0.76)

• student/pupil (0.72)

• age group 0 to 24 years (0.76)

• retired (0.80)

• couple with children (0.86)

• non-western immigrant (0.89)

The effects of these changes on the composition of the lowest SES group in 2001 can be judged in column 6 to 8.

The proportions in column 6 and 7 indicate the differences between the distribution across the sociodemographic categories within the lowest SES group as compared with the total population.

In the interpretation of our data it is important to make a distinction between the adjusted ratio of a category and the ratio of the lowest group compared with all respondents.

We illustrate this with the figures of the non-western immigrants. With an adjusted ratio of 0.89 their relative contribution to the lowest SES group has gone down between 1987 and 2001, however, in 2001 they are still overrepresented in the lowest SES group (8.9% in lowest SES group, 6.0% in total population)

In the age group 0 to 24 years the adjusted ratio in the lowest SES group was 0.76, at the same time the ratio in 2001 between the lowest SES group and all respondents was 0.65. In the age group 65 years and older these figures were 0.92 and 1.64 respectively, indicating that the relative contribution to the lowest SES group has gone down, but that there is still an overrepresentation of elderly in the lowest SES group.

As for *marital status*, the percentages of the divorced and the widowed in the lowest SES group were disproportionally high. The same applied to single households and single parent families in the category "*household distribution*". In the category "*employment status*" the unemployed, the disabled, housewife/men and the retired were overrepresented in the lowest SES group.

## Discussion

The aim of the current study was to analyse the changes within the lowest SES group that took place between 1987 and 2001. We hypothesised that the higher educational level might result in a smaller lowest SES group and that proportionally more persons with higher levels of education might reside in the lowest SES group and that this would apply in particularly for women.

### Methodological considerations

When using the same instrument over time, differences in the allocation of social class may be caused by societal developments. We chose occupation as SES indicator, because the raw data contained detailed information about the occupation of the respondents, whereas the available information about education was more basic. We had no information about income.

Comparisons of socioeconomic class and inequities in health over time are complicated by issues of measurement and adequacy of data. The cross-sectional design limits the determination of causal relationships.

A weak point in the current study was that for the allocation of SES we had to resort to the highest SES at household level or to the educational level in 31.1% of the cases in 1987 and 42.5% of the cases in 2001. This applies in particular for the younger age group. The group will on average demonstrate upward social mobility compared to the older cohort (their parents). This means that those who are still at school and do not have an own occupation yet, and who are assigned to the category according to their parents' occupation) will on average have their socioeconomic position underestimated.

We circumvented the problem of mixing socioeconomic status with educational level partly by excluding all respondents whose classification was based on educational level in the analysis of the relation between socioeconomic status and educational level.

In our study, like in most studies, an underestimation of changes is likely, due to an expected underrepresentation of certain less responding subgroups (non-western immigrants, unemployed, elderly), which are more prevalent in the lowest SES group.

### Summary of the results and explanation

The higher educational level did result in a smaller lowest SES group; it decreased in relative size from 34.9% to 29.4% between 1987 and 2001. This trend is visible not only in the present study, but all over Europe, although the size differs [14].

In the lowest SES group the proportion of women and men with high education has more than doubled between 1987 and 2001: in females the adjusted ratio was 2.96, in males it was 2.33. In females and males with a medium level of education the adjusted ratio was 2.19 and 1.64 respectively.

This indicates that the relation between educational level and occupation is less firmly anchored in 2001 than in 1987. The most probable explanation is that the increase in the number of persons with higher educational levels is higher than the growth of higher job categories with as consequence that more people with a higher education will end up in lower job categories. The assumption made in the introduction that females in particular were at risk for staying in an occupation-based low SES-group, was confirmed.

Obviously, this applies only for a minority of the higher educated; females with a high educational level represent 17.1% of all female respondents and only 43.4% of the females in the lowest SES group. In males a similar pattern is visible.

The presence of more persons with higher educational qualifications in the lowest SES group may influence the health status of the lowest SES group. A higher educational attainment is associated with lower levels of mortality, morbidity and a higher perceived health compared with a lower educational attainment [15]. Lahelma et al. (2004) demonstrated that inequalities by occupational class were largely explained by education [4]. Snittker et al. demonstrated that those with more education had better health for all levels of income, and that fewer income-based disparities existed among the well educated than among the less well educated [16]. Although we used occupation as socioeconomic indicator, the statistical association between occupation and income is strong enough to assume that Snittkers findings are relevant for our study.

Summarising, the increased proportion of higher educated in the lowest SES group, will most likely diminish the health inequalities between the lowest and higher SES groups, because higher educated persons have better health outcomes as compared with lower educated persons.

The relative proportion of young people in the lowest SES group decreased. This can be deducted from several sociodemographic subgroups. In the first place it can be read from the adjusted ratio in the age-group 0 to 24 years (0.76) But it can also be read from the adjusted ratios of males and females, who have not yet completed their education (0.73 and 0.67 respectively). Finally, the adjusted ratio of 0.72 for pupils and students in the category employment status points in the same direction. Besides the effect of a higher educational level, an additional explanation might be that in 2001 more youngsters were classified according to the head of the household than in 1987.

Another assumption made in the introduction was that proportionally less non-Western immigrants would reside in the lowest SES group when comparing 1987 and 2001, because of the higher educational achievement of the second generation of non-Western immigrants. This assertion proved to be justified, but the effect was limited; the adjusted ratio of non-Western immigrants was 0.89.

Despite the positive finding that the relative size of the lowest SES group decreases, we found that this did not apply to all sociodemographic categories; some categories fared worse, some fared better. Not all changes are important and need comment.

## Conclusion

Between 1987 and 2001a selective shift caused changes within the lowest SES group. What are the consequences of these mutations when comparing socioeconomic groups over time?

The most distinct change was that more persons with a higher educational level populated the lowest SES group. At the same time the relative proportion of some of the disadvantaged groups (divorced, unemployed) increased in the lowest SES group, but the size of this effect was smaller than the increase from higher education.

On balance, the changes in the composition did not result in an accumulation of disadvantaged groups in the lowest SES group. On the contrary, the influx of people with higher educational qualifications between 1987 and 2001 could result in better health outcomes and health perspectives of the lowest SES group.

## Abbreviations

DNSGP-1: first Dutch National Survey of General Practice

DNSGP-2 : second Dutch National Survey of General Practice

SES: socioeconomic stratum

## Competing interests

The author(s) declare that they have no competing interests.

## Authors' contributions

FB and LD were responsible for the design, for the analyses, and writing the article

FS participated in the data collection, analyses and the writing

HvdH and WvdB were responsible for the design and critically reviewed the article.

JvdZ supervised the data collection and critically reviewed the article

All authors approved the final manuscript.

## Pre-publication history

The pre-publication history for this paper can be accessed here:


